# Healing of Endosseous Implants Having Different Surface Characteristics in the Alveolar Bone: A Clinical Study

**DOI:** 10.7759/cureus.36990

**Published:** 2023-04-01

**Authors:** Khushboo Kumari, Kamal Nayan, Akshay Dinesh Joshi, Ishwariya Krishnan, Riddhi Sharma, Ravpreet Singh

**Affiliations:** 1 Department of Oral Pathology, Buddha Institute of Dental Sciences and Hospital, Patna, IND; 2 Department of Prosthodontics and Crown & Bridges, Mithila Minority Dental College and Hospital, Darbhanga, IND; 3 Department of Prosthodontics and Crown & Bridges, Mahatma Gandhi Vidya Mandir, Karmaveer Bhausaheb Hiray Dental College & Hospital, Nashik, IND; 4 Department of Oral Pathology and Microbiology, JKKN Dental College and Hospital, Erode, IND; 5 Department of Prosthodontics and Crown & Bridges, Baba Jaswant Singh Dental College, Hospital, and Research Institute, Ludhiana, IND

**Keywords:** turned surface, surface characteristics, oxidized surface, implant survival, dental implants, bone grafts

## Abstract

Background

Total treatment time in implant placement can be significantly reduced by placing immediate implants into the freshly extracted sockets. Also, immediate implant placement can act as a guide for proper and accurate implant placement. Additionally, in immediate implant placement, the resorption of bone associated with the healing of the extraction socket is also reduced. This clinical study aimed to clinically and radiographically assess the healing of endosseous implants having different surface characteristics in nongrafted and grafted bone.

Methodology

In 68 subjects, 198 implants were placed, including 102 oxidized (TiUnite, Göteborg, Sweden) and 96 turned surface implants (Nobel Biocare Mark III, Göteborg) were placed. Survival was considered with clinical stability and acceptable function with no discomfort and no radiographic or clinical signs of pathology/infection. Rest cases that showed no healing and implant no osseointegration were considered failures. Clinical and radiographic examination was done by two experts after two years of loading based on bleeding on probing (BOP) mesially and distally, radiographic marginal bone levels, and probing depth (mesial and distal).

Results

Five implants failed in total where four implants were with the turned surface (Nobel Biocare Mark III) and one was from the oxidized surface (TiUnite). The one oxidized implant was in a 62-year-old female and was placed in the region of mandibular premolar (44) of length 13 mm and was lost within five months of placement before functional loading. Mean probing depth had a nonsignificant difference between oxidized and turned surfaces with the mean values of 1.6 ± 1.2 and 1.5 ± 1.0 mm, respectively, with *P *= 0.5984; mean BOP in oxidized and turned surfaces was 0.3 ± 0.7 and 0.4 ± 0.6, respectively (*P *= 0.3727). Marginal bone levels, respectively, were 2.0 ± 0.8 and 1.8 ± 0.7 mm (*P *= 0.1231). In marginal bone levels related to implant loading, a nonsignificant difference was seen in early loading and one-stage loading with *P*-values of 0.06 and 0.09, respectively. However, in two-stage placement, significantly higher values were seen for oxidized surfaces (2.4 ± 0.8 mm) compared to turned surfaces (1.9 ± 0.8 mm), with *P *= 0.0004.

Conclusions

This study concludes that nonsignificantly higher survival rates are associated with oxidized surfaces compared to turned surfaces after two years of follow-up. Higher marginal bone levels were seen in oxidized surfaces for single implants and implants placed in two stages.

## Introduction

Total treatment time for implant placement can be significantly reduced by placing immediate implants into the freshly extracted sockets. Furthermore, immediate implant placement reduces bone resorption associated with extraction socket healing. However, recent literature data suggests no such effect is seen with immediate implant placement [1]. Typically, a gap is seen between the implant and extraction socket walls, with the largest gap in the coronal aspect of the socket [2]. Immediate dental implant placement is defined as inserting the implant into the socket immediately after dental extraction. Immediate implant placement has proven to be an acceptable procedure with predictable and successful long-term and short-term results. One of the major advantages of immediate implant placement is the elimination of the need to wait an additional three to six months following extraction for alveolar bone formation and the additional crestal bone loss that is associated with delayed implant placement. This bone loss is much less with immediate implant placement compared to delayed implant placement [2,3].

Animal studies revealed that osseointegration and the formation of new bone can close the horizontal gap formed between the implant and hard tissues if the gap is <1 mm. However, direct implant-to-bone contact is reduced when the gap is >1 mm. The concept of osseointegration in titanium implants was first introduced by Branemark et al. in 1969 and further explored in 1977 [4]. In the experimental findings, osseointegration is described as direct and close contact between implants and bone in the histological assessment. Clinically, however, osseointegration determines ankylosis and implant stability in the bone [5]. Albrektsson et al. proposed six factors that are considered critical to achieving acceptable osseointegration, including implant loading conditions, surgical technique, bone status, surface quality, implant design, and implant material [2]. Harmonious occlusion, good loading, a biocompatible implant surface, systemic health, initial implant stability, and bone width are all very important for successful implants [6,7].

Initially, two-stage surgery was recommended, with the mucoperiosteal flap reflected before abutments were placed, allowing for submerged implant healing [8]. Newer protocols with shorter healing times were suggested to reduce the management time for final prosthesis placement and eliminate the need for a second surgery. Modified surface topography implants were designed to promote early and improved bone healing [9]. However, rough implant surfaces are associated with many risks, including complications, peri-implant infections, and increased marginal resorption, as evidenced by previous literature data. For the posterior maxilla, bone augmentation can be replaced by sinus membrane lift during simultaneous implant placement without bone grafts [10]. This clinical study aimed to clinically and radiographically assess the healing of endosseous implants having different surface characteristics in nongrafted and grafted bone.

## Materials and methods

This clinical study aimed to clinically and radiographically assess the healing of endosseous implants having different surface characteristics in nongrafted and grafted bone. The study was conducted at Buddha Institute of Dental Sciences and Hospital, Patna, Bihar, India. Two implant surfaces (Nobel Biocare Mark III, Göteborg, Sweden, and TiUnite, Göteborg) with different surfaces were assessed for implant failure and compared for clinical efficacy of oxidized and turned implant surfaces after loading. A total of 68 subjects were included in the study after taking ethical clearance (ethical approval no. BIDSHR/2021/43). Nobel Biocare Mark III implants were placed in those subjects in the institute's Department of Oral and Maxillofacial Surgery. A total of 198 implants were implanted in 68 subjects, including 102 oxidized (TiUnite) and 96 turned surface implants (Nobel Biocare Mark III).

The inclusion criteria were subjects consenting to implant-supported fixed prostheses. While assessing efficacy after two years, five subjects died and 12 did not accept the invitation, leaving a final sample size of 51 subjects. The Branemark System protocol was followed for placing implants. Three experts in the field placed implants with high initial implant stability by selecting the final drill based on bone density. A staged protocol was followed in the study. Thirty-two subjects were managed with a one-stage protocol, 12 subjects were managed with early functional loading with early healing (mean 24 days), and the remaining with a delayed healing protocol (mean 17 weeks). Healing abutments were used during healing. After two weeks, a follow-up was done for suture removal, followed by a fixed prosthesis after 25 days in one-stage early loading.

Survival was considered possible with clinical stability and acceptable function, with no discomfort and no radiographic or clinical signs of pathology/infection, and bone levels were stable with no bone loss around the implant. Rest cases were regarded as a failure. Clinical and radiographic examinations were done by two experts after two years of loading based on bleeding on probing (BOP) mesially and distally, radiographic marginal bone levels, and probing depth (mesial and distal).

The collected data was subjected to statistical evaluation using SPSS, version 21 (IBM Corp., Armonk, NY, USA) and one-way analysis of variance (ANOVA) and t-tests for result formulation. Data was expressed in percentage, number, mean, and standard deviation. The level of significance was kept at *P *< 0.05.

## Results

The demographic characteristics of the study subjects are listed in Table 1. The mean age of the study subjects was 32.4 ± 4.68 years and the age range was 18 to 62 years. The study had 69.11% (*n *= 47) males and 30.88% (*n* = 21) females. In 68 subjects, 198 implants were placed, where 51.51% (*n *= 102) had an oxidized surface and 48.48% (*n *= 96) were turned surfaces. In the maxilla, 44.94% (*n *= 89) implants were placed, and in the mandible, 55.05% (*n *= 109) implants were placed (Table 1).

**Table 1:** Demographic data of the study subjects.

Characteristics	Number (*n*)	Percentage (%)
Gender		
Males	47	69.11
Females	21	30.88
Total subjects	68	100
Total implants placed	198	100
Implant surface		
Oxidized	102	51.51
Turned	96	48.48
Implant site		
Maxilla	89	44.94
Mandible	109	55.05

On assessing implant failure, five implants failed in total, where four implants were with the turned surface (Nobel Biocare Mark III) and one was from the oxidized surface (TiUnite). The oxidized implant was in a 62-year-old female and was placed in the region of mandibular premolar (44) of length 13 mm and was lost within five months of placement before functional loading. The other four lost implants were in subjects aged 45, 56, 61, and 60 years, at tooth positions 25, 22, 25, and 21 regions, respectively, three in females and one in males. The implant length, respectively, was 10, 15, 15, and 13 mm, and in all four subjects, one implant was lost in 10, 16, 6, and 6 months after implant placement, respectively, as shown in Table 2. 

**Table 2:** Implants lost and associated parameters in the study subjects.

Age (years)	Gender	Tooth position	Implant surface	Implant length (mm)	Implant lost (*n*)	Lost time (months)
45	Female	25	Turned	10	1	10
62	Female	44	Oxidized	13	1	5
56	Male	22	Turned	15	1	16
61	Female	25	Turned	15	1	6
60	Male	21	Turned	13	1	6

Concerning the assessment of implant healing parameters in the study subjects after two years, it was seen that the mean probing depth had a nonsignificant difference between oxidized and turned surfaces, with the mean values of 1.6 ± 1.2 and 1.5 ± 1.0 mm, respectively (*P *= 0.5984), and mean BOP in oxidized and turned surfaces was 0.3 ± 0.7 and 0.4 ± 0.6, respectively (*P *= 0.3727). Marginal bone levels, respectively, were 2.0 ± 0.8 and 1.8 ± 0.7 mm (*P *= 0.1231). Marginal bone levels concerning locations were assessed, and it was seen that in one-stage implants, it was significantly higher for the oxidized surface (1.6 ± 0.5 mm) compared to the turned surface (1.4 ± 0.4 mm), with *P *= 0.01. In the partial arch implants, levels were 2.3 ± 0.8 and 2.2 ± 1.1 mm, respectively (*P *= 0.5454). Similar nonsignificant levels were seen in full-arch implants with respective levels of 2.3 ± 1.1 and 2.1 ± 0.2 mm (*P *= 0.1425). In marginal bone levels related to implant loading, a nonsignificant difference was seen in early loading and one-stage loading having *P*-values of 0.06 and 0.09, respectively. However, in two-stage placement, significantly higher values were seen for oxidized surfaces (2.4 ± 0.8 mm) compared to turned surfaces (1.9 ± 0.8 mm), with *P *= 0.0004 (Table 3). 

**Table 3:** Implant healing parameters in oxidized and turned implant surfaces.

Location and type	Oxidized		Turned		*P*-value
	Mean ± SD	Range (Min-Max)	Mean ± SD	Range (Min-Max)	
Pocket depth (mm)	1.6 ± 1.2	0.9-5.6	1.5 ± 1.0	0.9-3.7	0.59
Bleeding on probing	0.3 ± 0.7	0.0-1.9	0.4 ± 0.6	0.0-1.9	0.37
Marginal bone levels	2.0 ± 0.8	0.1-5.4	1.8 ± 0.7	0.5-5.5	0.12
Single	1.6 ± 0.5	0.1-5.3	1.4 ± 0.4	0.5-5.5	0.01
Partial	2.3 ± 0.8	0.9-5.3	2.2 ± 1.1	1.0-5.5	0.54
Full-arch	2.3 ± 1.1	1.3-5.3	2.1 ± 0.2	1.3-2.4	0.14
Early loading	2.1 ± 0.4	1.5-2.8	2.2 ± 0.2	1.5-2.4	0.06
One-stage	1.9 ± 0.9	0.1-5.3	1.7 ± 0.4	0.7-2.1	0.09
Two-stage	2.4 ± 0.8	1.2-5.3	1.9 ± 0.8	0.5-5.5	0.00

## Discussion

Dental implant treatment is becoming popular and widely used as a result of recent advances in biomaterials and techniques for implant placement and stimulating bone regeneration in areas with deficient bone. Replacement of missing teeth with dental implants has shown predictable results with high success rates. As implants were first used, different ways to put them in and load them have been developed to make surgery easier and faster [11].

Schulte and Heimke first explained the immediate placement of dental implants in the extraction socket [12]. Immediate dental implant placement in the extraction socket has various advantages, including the maintenance of soft tissue aesthetics, alveolar bone preservation at the site of the tooth extraction, ideal placement of the dental implant in a three-dimensional spatial configuration, a shorter treatment period, and fewer surgical interventions [12]. However, there are some drawbacks to immediate implant placement, such as a lack of complete soft tissue closure over the extraction socket, thin tissue biotype, keratinized tissue absence, presence of periapical pathology, and side morphology.

This study assessed subjects with an age range of 18 to 62 years and a mean age of 32.4 ± 4.68 years. The study had more males than females. Oxidized and turned surface implants were placed in 51.51% (*n* = 102) and 48.48% (*n* = 96) of subjects, respectively. About 55.05% (*n* = 109) implants were placed in the mandible and 44.94% (*n* = 89) implants were placed in the maxilla. The demographics of this study were compared to the findings of Kim et al. [13] and Lee et al. [14], where subjects having comparable demographics were evaluated by the authors. One implant failed due to an oxidized surface (TiUnite), and four implants failed due to a turned surface (Nobel Biocare Mark III). The lost oxidized implant was from the mandibular premolar region, was 13 mm long, and was lost before functional loading in a 62-year-old female. The other four implants were lost in one male and three females and had a length of between 10 and 15 mm from the maxillary anterior and premolar regions. These studies were consistent with the studies by Chrcanovic et al. [15] and Buhara and Pehlivan [16], where the authors reported comparable implant failure due to bone and failure of osseointegration.

Implant healing parameters were assessed in the bone as well as soft tissues after two years of placement, showing nonsignificant probing depth, BOP, and marginal bone difference in the two groups (*P *= 0.5984, 0.3727, and 0.1231, respectively). Based on implant placement location, significantly higher marginal bone levels were seen for single implant placement on the oxidized surface than on the turned surface (*P* = 0.01). In marginal bone levels related to implant loading, a nonsignificant difference was seen in early loading and one-stage loading, with *P* = 0.06 and 0.09, respectively. However, in two-stage placement, significantly higher values were seen for oxidized surfaces (2.4 ± 0.8 mm) compared to turned surfaces (1.9 ± 0.8 mm), with *P* = 0.0004. These findings were in line with the studies by Chappuis et al. [17] and Kassim et al. [18], where similar bone and soft-tissue changes were suggested by the authors as in this study.

The few shortcomings of this study were the smaller number of subjects, a shorter assessment period, and geographical area biases requiring more studies to reach an absolute conclusion.

## Conclusions

In implant dentistry, attachment between the implant titanium surface and bone is a vital phenomenon. In implant design, a major consideration is achieving surfaces that promote desirable responses in tissues and bone. This can be achieved by surface modifying the implants. Oxidation of the surface has resulted in a higher survival rate and less bone loss surrounding the implant. Thus, this leads to the conclusion that oxidation can change surface characteristics, which positively affects osseointegration to have a greater implant success rate. 
